# Richmond and Louisville Medical Journal

**Published:** 1868-04

**Authors:** 


RICHMOND AND LOUISVILLE MEDICAL JOURNAL.—We would call the attention of our readers to the advertisement of the Richmond and Louisville Journal, to be found this month, in our advertisement sheet. We hope the liberal offer, to furnish the Richmond and Louisville Journal to all subscribers to the Buffalo Medical and Surgical Journal, for $3.00 per annum, will be accepted and appreciated. This Journal is edited with much labor and ability, and published at great expense, containing one hundred pages of the choicest and best-selected material. Prof. E. S. Gaillard, formerly of the Medical College of Virginia, now of the Kentucky School of Medicine, is the editor and proprietor, and does not fail to make every number of the Journal highly interesting and instructive.

